# The disposable bandage soft contact lenses therapy and anterior segment optical coherence tomography for management of ocular graft-versus-host disease

**DOI:** 10.1186/s12886-021-02031-0

**Published:** 2021-07-04

**Authors:** Yi-Chen Sun, Yoshihiro Inamoto, Ruikang K. Wang, Stephanie J. Lee, Kai-Feng Hung, Tueng T. Shen

**Affiliations:** 1 Department of Ophthalmology, Taipei Tzu Chi Hospital, The Buddhist Tzu Chi Medical Foundation, New Taipei City, Taiwan; 2 Department of Ophthalmology, University of Washington, 1959 NE Pacific St, Washington Seattle, USA; 3 College of Medicine, Tzu-Chi University, Hualien, Taiwan; 4 Clinical Research Division, Fred Hutchinson Cancer Research Center, Seattle, Washington USA; 5 Division of Hematopoietic Stem Cell Transplantation, National Cancer Center Hospital, Tokyo, Japan; 6 Department of Bioengineering, University of Washington, Seattle, Washington USA; 7 Department of Medical Research, Division of Translational Research, Taipei Veterans General Hospital, No.201, Sec 2, Shipai Rd., Beitou District, Taipei, Taiwan; 8 Department of Dentistry, School of Dentistry, National Yang-Ming Chiao Tung University, Taipei, Taiwan

**Keywords:** Ocular GVHD, OCT, Bandage soft contact lens

## Abstract

**Purpose:**

To identify the ocular surface changes of ocular graft-versus-host disease (GVHD) using anterior segment optical coherence tomography (AS-OCT) and examine the efficacy of disposable bandage soft contact lens (BSCL) treatment in ocular GVHD patients.

**Methods:**

This study is a prospective, Phase II clinical trial. Nineteen patients diagnosed with chronic GVHD based on the NIH criteria and ocular symptoms of NIH eye score 2 or greater were enrolled. Disposable BSCL was applied to the GVHD-affected eyes with topical antibiotic coverage. Ocular exams, eye symptom surveys, and AS-OCT were performed with signed informed consent. Patients were followed for one to three months.

**Results:**

Thirty-eight eyes of 19 patients with ocular GVHD underwent BSCL treatment in this study. AS-OCT scans were done in 14 out of 19 patients. The mean best-corrected visual acuity at enrollment, 2-week, and 4-week visits was 0.180, 0.128, and 0.163 logMAR, respectively. Twenty-four out of 25 eyes (96 %) that initially presented with conjunctival inflammation, twenty-three out of 30 eyes (76.7 %) that initially presented with punctate epithelial erosion, and 8 out of 15 (53.3 %) eyes that initially presented with filamentous keratopathy showed improvement after wearing BSCL for 2 to 4 weeks. AS-OCT revealed corneal epithelial irregularity, abnormal meibomian gland orifice, and conjunctival hyperemia, in patients with ocular GVHD.

**Conclusions:**

BSCL treatment provided significant subjective and objective improvements in ocular GVHD patients. Meanwhile, we found that AS-OCT can be a promising diagnostic tool to characterize the ocular surface changes associated with ocular GVHD.

**Supplementary Information:**

The online version contains supplementary material available at 10.1186/s12886-021-02031-0.

## Introduction

Graft-versus-Host Disease (GVHD) is a major complication of allogenic tissue/hematopoietic stem cell transplantation (allo-HSCT) [1]. GVHD can involve multiple organs after allo-HSCT. Ocular GVHD reportedly occurs in more than 50 % of allo-HSCT recipients with chronic GVHD [2] and significantly impairs vision-related quality of life of patients [3–5]. Clinically, the diagnosis and severity of ocular GVHD are primarily determined by the presence of ocular manifestations, such as new-onset dry, gritty, or painful eyes, keratoconjunctivitis sicca, photophobia, punctate keratopathy, and the positive Schirmer’ test [6]. However, the severity grading and diagnosis criteria may vary between institutes. For example, the NIH consensus proposed the NIH eye score as the ocular GVHD severity grading [7]. Japanese dry eye score is used in Japan for ocular GVHD diagnosis [8]. The international chronic ocular GVHD consensus group also proposed four subjective and objective variables – the ocular surface disease index (OSDI), Schirmer’s score without anesthesia, corneal staining, and conjunctival injection – for ocular GVHD diagnosis [9]. Thus far, an appropriate non-invasive ocular examination capable of characterizing the early changes of ocular GVHD has not been clearly defined.

Anterior segment optical coherence tomography (AS-OCT) is a non-invasive tool capable of examining most of the ocular structures in the anterior segment, including the cornea, conjunctiva, tear meniscus, and eyelid margin, with patients in an upright position. Since the first report by Izatt et al. in 1994 [10], the AS-OCT has become a promising imaging system for ocular disease diagnosis [11, 12]. In 2015, Li et al. employed AS-OCT to reveal the meibomian gland orifice obstruction, corneal epithelial irregularity, and prominent conjunctival lymphatic vessels in ocular GVHD patients [13]. However, only one patient was reported in this study. As such, a case series study using AS-OCT to examine the structural changes of ocular GVHD may constitute a basis for its use as a primary diagnostic or follow-up tool for this disease.

For the treatment of ocular GVHD, a variety of modalities have been reported, including artificial tear/ointment, punctal occlusion [14], allogeneic serum eye drops [15], cyclosporine eyedrops [16, 17], autologous platelet lysate [18], and tacrolimus ointment [19]. In addition to topical medications, the scleral lens [20] and bandage soft contact lens (BSCL) [21] were also used for ocular GVHD patients. Several previous studies showed that the prosthetic replacement of the ocular surface ecosystem (PROSE) lenses, a type of scleral lens, was effective in mitigating symptoms and resurfacing corneal erosions of severe ocular GVHD [20, 22]. However, PROSE lens is high cost and made on-site only at the Boston Foundation for Sight, Needham, MA, USA, making PROSE lens less accessible to all patients. On the other hand, BSCL seems to be an ideal alternative for the treatment of ocular GVHD. BSCL is available in different diameters with a range of base curves, can be fitted and dispensed on the same day, and is less expensive than PROSE lenses. Importantly, BSCL was reported to provide appropriate protection of ocular surface from inflamed palpebral conjunctiva [21].

While BSCL treatment is associated an improvement of symptoms, the ultra-structural changes in these ocular GVHD patients have not been clearly characterized. Therefore, the objective of this Phase II clinical trial is two-fold: to evaluate the treatment efficacy of BSCL and the potential of AS-OCT as a diagnostic or follow-up tool for ocular GVHD, aiming to assess the value of combining BSCL and AS-OCT for the management of ocular GVHD patients.

## Materials and methods

### Study design and patient eligibility

All the patients were evaluated at enrollment for their systemic and ocular GVHD by transplant clinicians and ophthalmologists, respectively. Patients were eligible to recruit into this study if they were age 18 years or above, met the diagnosis of chronic GVHD per NIH criteria, had moderate to severe ocular GVHD, and did not have new systemic immunosuppressive medications within one month prior to enrollment. The OSDI-based symptom questionnaire was completed at enrollment, 2 weeks, 4 weeks, and 3 months after BSCL placement. The results of the best-corrected visual acuity (BCVA), slit-lamp examination, and AS-OCT were collected at the enrollment, 2-week, and 4-week visits. The setting of AS-OCT and the exclusion criteria have been previously described [13, 23]. This study was approved by the Institutional Review Board of Fred Hutchinson Cancer Research Center and registered at www.clinicaltrials.gov as NCT01616056 on 11/06/2012. All patients signed informed consent documents.

### Disposable bandage soft contact lens (BSCL) application

Silicone hydrogel BSCL for the eyes of ocular GVHD-affected patients was selected based on its anterior corneal curvature. All patients wore the BSCL for 24 h a day. Four different brands were used in this study, including PureVision (Bausch & Lomb, Rochester, NY), SofLens 38 (Bausch & Lomb, Rochester, NY), Flexlens (Ideal Optics, Duluth, GA), and Kontur (Kontur Kontact Lens, Hercules, CA). The PureVision BSCL with 14.0-mm diameter and 0.05-mm center thickness was the first choice for patients. If patients were bothered by foreign body sensation, SofLens 38 contact lens with 0.038-mm center thickness was used. For patients unable to accommodate SofLens 38 contact lens, Flexlens and Kontour BSCL (with 15.0-mm diameter and 0.1-mm thickness) were used. BSCL with extended wear was replaced every two weeks at the ophthalmology clinic during the study. The patients were instructed to use antibiotic eye drops (Ofloxacin 0.3 % ophthalmic solution or Moxifloxacin HCL 0.5 % ophthalmic solution), as well as the topical lubricants, including artificial tears, gels, and ointments, four times per day.

### Outcome assessment and Statistical Analysis

The symptom questionnaires (see supplementary material) were completed by patients at enrollment and during follow-up visits. Questions related to ocular symptoms included the “1. Bothered by: dry eyes”, “2. Bothered by: need to use eye drops frequently”, “3. Bothered by: difficulty seeing clearly”, “4. Experienced: eyes that are sensitive to light”, “5. Experienced: eyes that feel gritty”, “6. Experienced: painful or sore eyes”, “7. Experienced: blurred vision”, “8. Experienced: poor vision”, “9. Limited performing: reading”, “10. Limited performing: driving at night”, “11. Limited performing: working with a computer or bank machine (ATM)”, “12. Limited performing: watching TV”, “13. Uncomfortable situation: windy conditions”, “14. Uncomfortable situation: places or areas with low humidity (very dry)”, and “15. Uncomfortable situation: areas that are air-conditioned”. The baseline, 2-week, and 4-week ocular assessments included the visual acuity measurement and slit-lamp examination. A LogMAR value of 0 was equivalent to 20/20 vision on a Snellen chart. The BCVA was converted to the logMAR scale for statistical analysis. Corneal filaments (absence or presence) and punctate epithelial erosions (none, 1+, 2+, 3+, 4+) were assessed by the slit-lamp exam. The continuous data (visual acuity) used mixed model analysis approaches. Symptom data were analyzed by McNemar test. The data of corneal filaments and punctate epithelial erosions were analyzed using Kruskal-Wallis test.

## Results

### Patient Demographics

Nineteen patients were included in this study (Table 1). The average age of the patients was 55 years old (range, 32–75 years old). The underlying diagnosis of these patients included acute leukemia (7 cases, 37 %), chronic leukemia (2 cases, 11 %), malignant lymphoma (5 cases, 26 %), and myelodysplastic syndrome (5 cases, 26 %). Fourteen patients (74 %) had moderate ocular GVHD, and 5 (26 %) had severe ocular GVHD at enrollment. These patients had been treated with artificial tears, viscous ointment, cyclosporine eye drops, flax seed oil, punctal plugs, steroid eye drops, occlusive eyewear, or Cevimeline at or before enrollment.

**Table 1:** Patient characteristics at study enrollment (*N* = 19)

Variable	Value
Age (years), median (range)	55 (32–75)
Patient gender, no. (%) Male Female	11 (58) 8 (42)
Transplant to study entry (months), median (range) Ocular GVHD diagnosis to study entry (months), median (range)	36.5 (8.0-157) 7.0 (1.0-133)
Diagnosis, no. (%) Acute leukemia Myelodysplastic syndrome Malignant lymphoma Chronic leukemia	7 (37) 5 (26) 5 (26) 2 (11)
Donor-patient gender combination, no. (%) Female to male Other	4 (21) 15 (79)
HLA and donor type, no. (%) Identical sibling Matched unrelated Mismatched unrelated	5 (26) 12 (63) 2 (11)
NIH eye score at the enrollment, no. (%) Moderate (score 2) Severe (score 3)	14 (74) 5 (26)

### The characteristics of ocular GVHD revealed by anterior segment optical coherence tomography (AS-OCT)

**Fig. 1:**
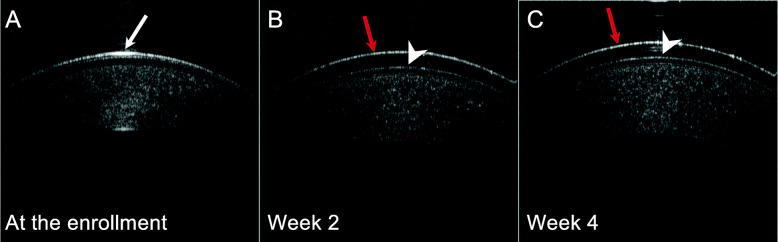
Representative AS-OCT imaging of cornea photographs from one ocular GVHD at the enrollment, week 2, and week 4. **A** Apparent corneal epithelial irregularity (white arrow) was noted at the enrollment. **B** & **C** Less corneal epithelial irregularity (white arrowhead) was found after 2 and 4 weeks of the BSCL placement. The red arrow indicates bandage soft contact lens

**Fig. 2:**
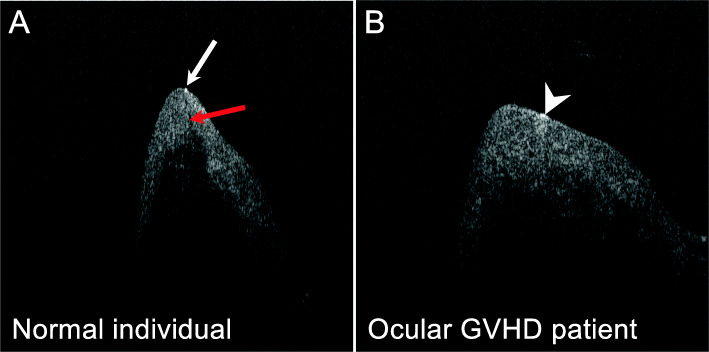
Ocular GVHD patients with meibomian gland orifice obstruction. **A** Representative AS-OCT of the eyelid margins of a healthy individual. **B** Representative AS-OCT of the eyelid margins of an ocular GVHD patient. The red arrow indicates the meibomian gland lumen, and the white arrow indicates a small plug over the meibomian gland orifice. The white arrowhead indicates a plug of the meibomian gland without a visible gland lumen

**Fig. 3:**
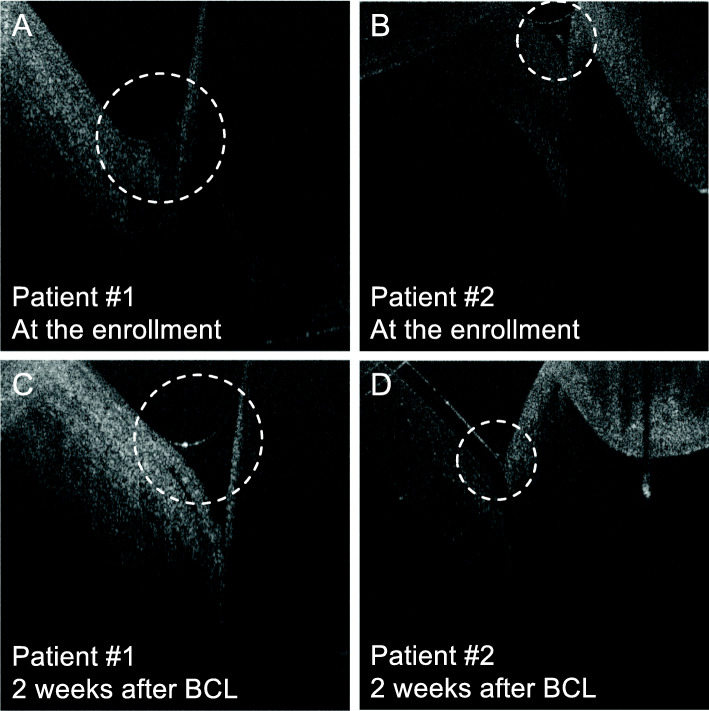
Representative AS-OCT imaging of the tear menisci between the lower eyelid and inferior bulbar conjunctiva. **A** & **B** The tear meniscus of ocular GVHD patients #1 was turbid in appearance, whereas the tear meniscus of patient #2 was clear in appearance at the enrollment. **C** & **D** Two weeks after the BSCL treatment, the height of tear meniscus of patient #1 was not decreased, whereas the height of tear meniscus of patient #2 was decreased

Among 19 patients enrolled in the clinical trial, 14 patients received AS-OCT image examination at the enrollment and during the follow-up period (5 patients missed the follow-up AS-OCT imaging due to an equipment issue). All of the 14 patients had corneal epithelial irregularity prior to the treatment (Fig. 1). Two weeks after BSCL placement, a decrease in corneal epithelial irregularity was noted in 12 of these patients. Among those with a reduced corneal epithelial irregularity, 75 % (9/12) of patients also had improved vision. On the other hand, obstruction of meibomian gland orifices was observed at the enrollment and remained unchanged during the follow-up visits (Fig. 2). In addition, the tear meniscus assessed by AS-OCT was found to be increased in 6 patients and decreased in four patients, while another four patients did not show significant change after 2–4 weeks of BSCL treatment. The tear meniscus can be turbid (Fig. 3A) or clear (Fig. 3B) in appearance prior to the BSCL treatment, and the height of the tear meniscus was found to be unchanged (Fig. 3C) or decreased (Fig. 3D) after prolonged BSCL treatment.

### Bandage soft contact lens (BSCL) treatment improves visual acuity and ocular symptoms of patients

**Fig. 4:**
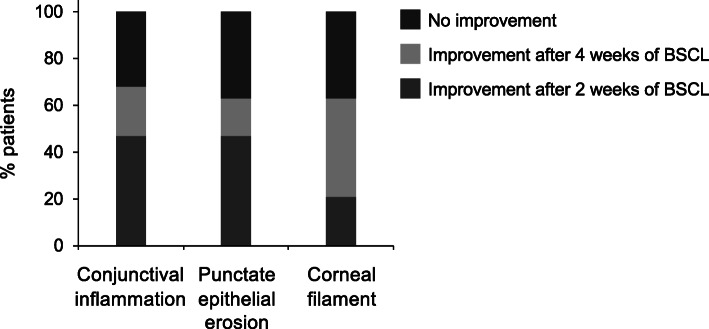
Ocular findings of ocular graft-versus-host disease patients collected at enrollment, two weeks, and four weeks after BSCL treatment

The mean BCVA logMAR values at the enrollment, 2-week, and 4-week visits were 0.180 ± 0.176, 0.128 ± 0.159, and 0.163 ± 0.194, respectively. There was a significant improvement between 2-week and initial visits (*P* = 0.023). Intriguingly, the improvement of BCVA seems to be less significant four weeks after treatment (*P* = 0.915) (Table 2). Meanwhile, the ocular symptoms, including conjunctival inflammation, punctate epithelial erosion, and corneal filament, were also analyzed after BSCL treatment. Among 19 ocular GVHD patients, 9 (47 %) and 4 (21 %) patients had an improvement of conjunctival inflammation after 2 and 4 weeks of treatment, respectively, whereas the other 6 (32 %) patients continued to exhibit conjunctival inflammation after treatment. Regarding the clinical presentations, 9 (47 %) and 3 (16 %) out of 19 patients presented less punctate epithelial erosions after 2 and 4 weeks of BSCL treatment, respectively. Moreover, 4 (21 %) and 8 (42 %) out of 19 patients were found to have an improvement in the appearance of corneal filament after 2 and 4 weeks of BSCL treatment, respectively (Fig. 4).

**Table 2:** Visual acuity of ocular graft-versus-host disease patients at the enrollment, 2-week, and 4-week bandage soft contact lens treatment. * indicates *P* < 0.05

	LogMAR	Mean Snellen	Change from baseline
Baseline	0.180 ± 0.176	20/31	NA
Week 2	0.128 ± 0.159	20/27	0.023 *
Week 4	0.163 ± 0.194	20/29	0.915

### OSDI-based symptom assessment after bandage soft contact lens treatment

The OSDI-based symptom questionnaire, including 15 survey questions was completed by 19 patients at the enrollment, two weeks, four weeks, and three months after BSCL treatment. The patients reported a significant improvement in question 3 (Bothered by: difficulty seeing clearly) of the questionnaire at week-2 (*p* = 0.031), question 5 (Experienced: eyes that feel gritty) of the questionnaire at week-2, week-4, and week-12 (*p* = 0.008, 0.002, 0.031, respectively), and question 6 (Experienced: painful or sore eyes) at week-2 and week-4 (*p* = 0.002 and 0.004, respectively). In other words, the patients who experienced difficulty seeing, gritty, and/or painful eyes benefited the most from BSCL treatment.

## Discussion

This Phase II clinical trial showed the excellent outcomes of disposable BSCL treatment for ocular GVHD-affected eyes without any significant complication. The symptom questionnaire showed significant improvement in patients’ gritty and painful sensations after BSCL treatment. The vision of patients was also significantly improved after two weeks of treatment. The AS-OCT scan at the enrollment revealed meibomian gland orifice obstruction and significant corneal epithelial irregularity of the ocular GVHD-affected eyes. After the BSCL treatment, the corneal epithelial irregularity was improved as assessed by AS-OCT, thus suggesting that the value of AS-OCT in monitoring the progress of ocular GVHD.

To date, the optimal approach for early diagnosis and follow-up of ocular GVHD has not been defined. Indeed, while questionnaire-based diagnostic or grading criteria, including the NIH eye score, Lee eye subscale, OSDI, and Schirmer test, showed high sensitives for ocular GVHD, a definitive diagnosis of ocular GVHD often requires the pathologic or cytologic reports. In addition, the selection of an appropriate sampling approach also varies between studies. Although conjunctival scrape cytology or biopsy yields a sufficient number of cells for diagnosis, these approaches are invasive [25, 26]. On the other hand, AS-OCT is a non-invasive tool for ocular structure assessment. AS-OCT can be useful for the evaluation of various ocular structures, such as cornea [24–26], conjunctiva [27], sclera [28], iris [29], and angle structures [30, 31]. Because ocular GVHD is mainly presented in the anterior part of the eye, AS-OCT should be of value for assessing and grading ocular GVHD.

In this study, AS-OCT revealed the obstruction of the meibomian gland orifice in most of the ocular GVHD patients, suggesting that the morphology of meibomian glands on OCT imaging may be useful to detect the onset of ocular GVHD. Dysfunction or loss of the meibomian gland has been regarded as a mechanism contributing ocular symptoms of GVHD patients [32], and meibomian gland orifice obstruction could be observed in post-HSCT patients [27]. Previously, a slit-lamp examination was the primary method for evaluating the meibomian gland orifices in vivo. Noncontact infrared meibography and in vivo laser scanning confocal microscopy (IVCM) were then used to detect the morphology of meibomian glands, the acinar density, and diameter and gland orifice features [33–35]. However, IVCM is limited by its cross-sectional scanning mode and working distance. Therefore, AS-OCT seems to be a reasonable device for the assessment of meibomian glands and diagnosis of ocular GVHD. Additionally, we used AS-OCT and identified corneal epithelial irregularity in all of the trial patients. Importantly, 12 out of 14 patients were shown to present less corneal epithelial irregularity after BSCL treatment, highlighting the value of AS-OCT for the evaluation of ocular GVHD. Our AS-OCT also revealed that the height of tear meniscus was not consistently decreased in ocular GVHD patients, and the patients with improved ocular symptoms may not necessarily exhibit an increase in tear meniscus.

For the ocular GVHD patients, while the first-line therapy remains the medication, the intervention therapy is growing in popularity. The drug of choice includes the topical immunosuppressant, anti-allergic drugs, allogeneic serum eye drops, and autologous plasma rich in platelet-derived growth factor (PDGF) eye drops [15, 18, 23]. For medication-refractory patients, intervention therapy is a vital treatment option [14, 21]. Among them, disposable BSCL is less expensive and more acceptable to patients than the other rigid contact lens. The disposable BSCL has been applied to a variety of ocular surface diseases, such as superior limbic keratoconjunctivitis, neurotrophic keratopathy [36–39]. A previous study and our studies also showed an improvement of ocular symptoms following BSCL treatment [21]. BSCL functions as a barrier between palpebral conjunctiva and cornea to promote surface protection and the re-epithelialization of the corneal surface, leading to an improvement of corneal irregularity and relief of ocular symptoms. Importantly, we used AS-OCT and found that 12 out of 14 patients presented with less corneal epithelial irregularity after BSCL treatment, highlighting the value of BSCL and AS-OCT for the treatment and evaluation of ocular GVHD.

While we showed that the outcome of disposable BSCL treatment and the diagnostic efficacy of AS-OCT is promising, there are still several limitations of our study. One limitation relates to the fact that it is essentially not possible to conduct a double-masked BSCL clinical trial. Nonetheless, in this study, the ocular symptoms and AS-OCT of trial patients were separately examined by two reviewers, and the interpretations of ocular symptoms and AS-OCT are mostly consistent between independent reviewers. Another limitation is that this study did not include a group of control patients. However, as the natural course of topical lubricant application to ocular GVHD patients has been extensively characterized, and the safety of BSCL treatment has already been proven, this clinical trial was designed as a single-arm study.

In conclusion, BSCL that acts as a barrier between the ocular surface and inflamed palpebral conjunctiva can improve subjective symptoms and objective ocular findings of patients, thus proven to be an effective treatment for ocular GVHD patients. Meanwhile, AS-OCT can reveal the meibomian gland orifice obstruction and corneal epithelial irregularity, which represent the key features of ocular GVHD at the ultrastructural level. Because the GVHD-associated ocular symptoms may be irreversible and potentially vision-threatening, AS-OCT that is non-invasive and can be performed repetitively serves as a useful tool for early recognition prior to the onset of severe complications.

## Supplementary Information

**Additional file 1.**

## Data Availability

The datasets used and analyzed during the current study available from the corresponding author on reasonable request.
