# Correction to: The disposable bandage soft contact lenses therapy and anterior segment optical coherence tomography for management of ocular graft-versus-host disease

**DOI:** 10.1186/s12886-021-02225-6

**Published:** 2022-01-25

**Authors:** Yi-Chen Sun, Yoshihiro Inamoto, Ruikang K. Wang, Stephanie J. Lee, Kai-Feng Hung, Tueng T. Shen

**Affiliations:** 1 College of Medicine, Tzu-Chi University, Hualien, Taiwan; 2 Department of Ophthalmology, Taipei Tzu Chi Hospital, The Buddhist Tzu Chi Medical Foundation, New Taipei City, Taiwan; 3 Department of Ophthalmology, University of Washington, 1959 NE Pacific St, Seattle, WA USA; 4 Clinical Research Division, Fred Hutchinson Cancer Research Center, Seattle, WA USA; 5 Division of Hematopoietic Stem Cell Transplantation, National Cancer Center Hospital, Tokyo, Japan; 6 Department of Bioengineering, University of Washington, Seattle, WA USA; 7 Department of Medical Research, Division of Translational Research, Taipei Veterans General Hospital, No.201, Sec 2, Shipai Rd., Beitou District, Taipei, Taiwan; 8 Department of Dentistry, School of Dentistry, National Yang-Ming Chiao Tung University, Taipei, Taiwan

**Correction to: BMC Ophthalmol 21, 271 (2021)**

**https://doi.org/10.1186/s12886-021-02031-0**

Following the publication of the original article [1], we were notified that an affiliation was missed for Yi-Chen Sun: College of Medicine, Tzu-Chi University, Hualien, Taiwan.

The original article has been corrected.
