# Supplementary material for: The disposable bandage soft contact lenses therapy and anterior segment optical coherence tomography for management of ocular graft-versus-host disease
Source: BMC Ophthalmol. 2021 Jul 4;21:271. doi: 10.1186/s12886-021-02031-0 (PMC8254955; doi:10.1186/s12886-021-02031-0)
Supplement: Supplementary file 1 — Additional file 1. [file 12886_2021_2031_MOESM1_ESM.pdf]

Bandage Lenses Study Patient Survey

FOLLOW-UP

INSTRUCTIONS

This survey will provide us with important information about your health.

All your answers will be kept strictly confidential and will not be included in your medical record. The information that you provide will be combined with that of many other transplant patients before analysis.

Please read each question carefully. Circle or check off the answer that best describes how you feel.

While we ask that you answer each question, you are free to *not* answer any question that makes you feel uncomfortable. If none of the answers provided seems exactly right, choose the one that comes closest to being right for you. Some of the questions may seem the same. However, it is important that we ask about certain aspects of your health in different ways in order to fully understand how you are feeling.

When you have completed this survey, please give it back to the study coordinator or mail it back to us using the enclosed self-addressed, stamped envelope.

We greatly appreciate your participation.

Your name: ________________________ Date: ________________

Section 1: Your Chronic Graft vs. Host Disease (GVHD) Symptoms

Please circle the number that shows how severe your symptoms have been **in the last week**:

		Not Present										As Bad As You Can Imagine
1.	Your chronic GVHD symptoms **overall**?	0	1	2	3	4	5	6	7	8	9	10
2.	Your **eye** problem at its WORST?	0	1	2	3	4	5	6	7	8	9	10

Compared to before you had lenses, how would you rate your following GVHD symptoms now?

		Compl-etely gone	Very much better	Moder-ately better	A little better	About the same	A little worse	Moder-ately worse	Very much worse
1.	**GVHD symptoms overall**	1	2	3	4	5	6	7	8
2.	**Eye**	1	2	3	4	5	6	7	8

What are your reasons for saying your chronic GVHD is better or worse overall? (Is there a symptom of particular concern to you that has changed?)
(Write in)

____

____

____

Section 2

By circling one (1) number per line, please indicate how much you have been bothered by the following problems in the past month:

**EYES:**		**Not at all**	**Slightly**	**Moderately**	**Quite a bit**	**Extremely**
1.	Dry eyes…………………………….	0	1	2	3	4
2.	Need to use eye drops frequently..	0	1	2	3	4
3.	Difficulty seeing clearly…………..	0	1	2	3	4

Section 3

Have you experienced any of the following during the last week?		All of the time	Most of the time	Half of the time	Some of the time	None of the time	
1.	Eyes that are sensitive to light?....................................	4	3	2	1	0	
2.	Eyes that feel gritty?.............	4	3	2	1	0	
3.	Painful or sore eyes?............	4	3	2	1	0	
4.	Blurred vision?......................	4	3	2	1	0	
5.	Poor vision?..........................	4	3	2	1	0	

Have problems with your eyes limited you in performing any of the following during the last week?		All of the time	Most of the time	Half of the time	Some of the time	None of the time	Not applicable
6.	Reading? ……………………..	4	3	2	1	0	N/A (-2)_
7.	Driving at night? …………….	4	3	2	1	0	N/A (-2)_
8.	Working with a computer or bank machine (ATM)? ………	4	3	2	1	0	N/A (-2)_
9.	Watching TV? ……………….	4	3	2	1	0	N/A (-2)_

Have your eyes felt uncomfortable in any of the following situations during the last week?		All of the time	Most of the time	Half of the time	Some of the time	None of the time	Not applicable
10.	Windy conditions?.................	4	3	2	1	0	N/A (-2)_
11.	Places or areas with low humidity (very dry)?..............	4	3	2	1	0	N/A (-2)_
12.	Areas that are air conditioned?..........................	4	3	2	1	0	N/A (-2)_

Other health-related questions

**Did you see another eye doctor during this interim?**	❑ Yes (1)	❑ No (0)
**If yes,** ask contact information and get the record.		

**Adverse events (any events not necessarily relevant to eyes)**		
1. Have you experienced any medical problems since your last visit for the study?	❑ Yes (1)	❑ No (0)
**If yes,** describe when and what kind of adverse events.		
2. Do you feel like there is something in your eye?	❑ Yes (1)	❑ No (0)
3. Are your eyelids swollen?	❑ Yes (1)	❑ No (0)
4. Do you have excessive tearing?	❑ Yes (1)	❑ No (0)

**Compliance**		
1. Have you been wearing your bandage lenses all the time?	❑ Yes (1)	❑ No (0)
If No, when did you stop it? And why?		
2. Have you been using the antibiotic drops as recommended?	❑ Yes (1)	❑ No (0)
If No, when did you stop it? And why?		

**Current Treatment of Eye GVHD** **(Check all that apply)** **Please compare with the enrollment form.**	**Treatment**	
	Artificial tears (1)	❑
	Slow release artificial tears (Lacriserts) (2)	❑
	Viscous ointment (i.e.. Refresh PM, Celluvisc) (3)	❑
	Restasis (4)	❑
	Steroid eye drop (5)	❑
	Autologous serum tears (6)	❑
	Punctal plugs (7)	❑
	Punctal cauterization (8)	❑
	Flax seed oil (9)	❑
	Evoxac (cevimeline) (10)	❑
	Occlusive eye wear (11)	❑
	Moisture chamber eye wear (12)	❑
	Other ________________ (13)	❑

**Current medications' dose regimens**			
Medication change	❑ Yes (1)	❑ No (1)	*If yes, indicate when and which medication(s).*

Thank you for participating in this study

Please remember that someone is available to speak with you at any time, if you wish.
Dr. Stephanie Lee may be reached by calling (206) 667-5160. She will be able to answer any questions about the study or refer you to other support staff as needed.

Please use the space below for any other comments.

For office use only:

Study ID	*Initials (First, Last)*	*Date completed:*	*Date received:*
Timepoint: *V3.0*		*Date entered:*	
